# Regulation of fibroblast-like synoviocyte function by cadherin 6 in rheumatoid arthritis

**DOI:** 10.1186/s13075-025-03637-1

**Published:** 2025-08-29

**Authors:** Camilla R. L. Machado, Eunice Choi, Narayanan B. Perumal, Robert J. Benschop, Gregory R. Dressler, Wei Wang, David L. Boyle, Gary S. Firestein

**Affiliations:** 1 Division of Rheumatology, Autoimmunity and Inflammation, UC San Diego School of Medicine, San Diego, CA 92093 USA; 2 Department of Chemistry and Biochemistry, UC San Diego, San Diego, CA 92093 USA; 3 Eli Lilly and Company, Indianapolis, IN 46285 USA; 4 Department of Pathology, University of Michigan, Ann Arbor, MI 48109 USA

## Abstract

**Background:**

Cadherins (CDH), such as CDH11, are glycoprotein adhesion molecules contributing to cell-cell interactions in health and disease. CDH11 has demonstrated important functions in rheumatoid arthritis (RA) fibroblast-like synoviocytes (FLS). In transcriptome expression studies, we observed that Cadherin 6 (CDH6) expression was higher in RA compared to osteoarthritis (OA). CDH6 is associated with cancer progression, but little information is known on the role of CDH6 in RA. The present study investigates CDH6 expression, regulation, function in FLS, and distribution in RA synovia.

**Methods:**

Synovial tissue and FLS were obtained from RA or OA patients undergoing joint replacement. CDH6 epigenetic marks and expression in RA and OA FLS were evaluated using public databases. CDH6 expression was determined by RT-PCR, Western blot, and immunostaining. RA and OA FLS were stimulated with cytokines and growth factors, and CDH6 mRNA expression was determined. CDH6 was silenced using siRNA, and the effect on migration, cell growth, apoptosis, autophagy, cell cycle, and signaling was studied.

**Results:**

In our analysis of cadherin family expression, CDH6 expression was higher in RA than OA FLS. This was associated with differential chromatin accessibility and histone marks in the CDH6 promoter of RA FLS. H3K27ac was identified as an important regulator of CDH6 expression in RA FLS based on experiments using histone deacetylase inhibitors. TGFß, but not IL-1β, TNF, IL-17A, IFNγ, IL-6, or PDGF, increased CDH6 expression of cultured RA FLS. CDH6 knockdown significantly decreased RA FLS migration and cell growth. The latter was associated with increased apoptosis in CDH6 deficient FLS. Immunofluorescence showed CDH6 protein distribution in the membrane, perinuclear, and nuclear regions of cultured FLS. In RA synovial tissue, CDH6 expression was noted in FLS and macrophages within the lining and sublining regions.

**Conclusions:**

CDH6 expression is elevated in RA FLS due to epigenetic and local conditions of synovitis promoting migration, survival and cell growth, which are characteristic features of aggressive RA FLS. The intracellular distribution suggests additional functions beyond adhesion and homotypic aggregation, such as signaling and gene regulation. These data suggest CDH6 contributes to RA pathogenesis by influencing pathologic FLS behavior and could be a therapeutic target.

**Supplementary Information:**

The online version contains supplementary material available at 10.1186/s13075-025-03637-1.

## Introduction

Rheumatoid arthritis (RA) is a chronic immune-mediated disease characterized by synovial inflammation and joint destruction. Fibroblast-like synoviocytes (FLS) are mesenchymal cells that regulate synovial inflammation and display an aggressive phenotype in RA [1–4]. Expression of cell adhesion molecules like cadherins by FLS have been implicated in this phenotype, especially cadherin 11 (CDH11) [5–8]. The cadherin family has diverse biological roles that could have broader functions relevant to RA because they regulate cell-cell adhesion and cell signaling, play a role in organ morphogenesis and tissue homeostasis, and are associated with cancer [5, 6, 9]. However, the contribution of cadherins other than CDH11 toRA pathology is unclear.

To understand the potential role of cadherins in RA, we profiled CDH expression in RA and osteoarthritis (OA) using our previously reported transcriptome data. Cadherin 6 (CDH6) emerged as a gene of interest based on high expression in RA compared with OA. It is a member of the type II cadherin family with a role in organ development, particularly the kidney, and has been associated with tumor progression and metastasis in malignant cells. It has been implicated in several types of carcinoma, such as renal, ovarian, thyroid, and lung cancers [5, 10–13]. CDH6 has five extracellular domains and a cytoplasmic domain, with His-Ala-Val motif and RGD motifs, suggesting that it not only regulates cell-cell interactions but also cell-matrix interactions [14]. Because of these features, CDH6 is being evaluated as a target for biotherapeutics development in cancer [15, 16]. However, its function in RA and its contribution to synovial pathology are unknown.

In this study, we focused on the dysregulation of CDH6 in RA and considered its potential as a novel treatment target. Our findings indicate that increased CDH6 expression in RA FLS compared to OA FLS is associated with epigenetic modifications in the CDH6 promoter, particularly through histone modification. Furthermore, CDH6 promotes RA FLS growth, survival, and migration. In addition, we revealed CDH6 presence in the membrane, cytoplasm, and, notably, the nucleus, suggesting potential roles beyond cell adhesion. These results suggest that CDH6 plays a critical role in RA pathogenesis, representing a possible therapeutic target for modulating aggressive FLS behavior. Because CDH6 does not appear to participate in adaptive immune responses, targeting this protein could potentially be used with immunosuppressive agents.

## Methods

### Synovial tissue and human FLS culture

Synovial tissue was obtained from RA or OA patients during their joint replacement surgery or synovectomy. The Human Research Protection Program approved the protocol and all patients provided informed consent (Protocol #14–0175). RA patients met the American College of Rheumatology 2010 criteria [17], and for OA, the diagnosis conformed to the 1991 criteria [18]. FLS were obtained from synovial tissues as previously described and used from passages 4 through 7 [19]. Cells were cultured in Gibco Dulbecco’s Modified Eagle Medium (DMEM) supplemented with L-glutamine, penicillin/streptomycin, and gentamicin (complete DMEM), and 10% heat‐inactivated Fetal Calf Serum (FCS) at humidified 5% CO2 atmosphere. For all experiments, FLS were serum-starved with 1% fetal bovine serum (FBS), followed by replacement with fresh 1% FBS medium for subsequent experiments. Clinical correlations were not possible because the biosamples were de-identified.

### RNAseq, ATACseq and ChIPseq data analysis

RNA sequencing (RNAseq), and epigenetic analysis were performed using our previous epigenetic landscape data (Gene Expression Omnibus with the primary accession code GSE112658) [20] using DiffBind package in R (FDR < 0.05) for ATACseq and ChIPseq between RA and OA FLS [21]. The quantification of gene and isoform analysis used RSEM (version 1.3.1).

### Real-time quantitative polymerase chain reaction (RT-qPCR)

Isolation of total RNA from synovial tissues and FLS was done using RNeasy mini kit (Qiagen) following the manufacturer’s instruction. The complementary DNA (cDNA) was prepared from 250ng of RNA using TaqMan reverse transcription reagents (Thermo Fisher Scientific), and qPCR was performed using primer and probe set (Thermo Fisher Scientific) and StepOne™ Real-Time PCR System (Thermo Fisher Scientific). Resulting threshold C(t) data were normalized to GAPDH expression using standard curves constructed from serial dilutions of cDNA derived from human FLS under IL-1β stimulation, yielding cell equivalents. The ratio between the gene of interest and GAPDH cell equivalents was expressed as relative expression units (REU).

### Gene Silencing

FLS were transfected with 1 µg of CDH6 small interfering Smart Pool On-Target RNA. (siRNA), Horizon Discovery) or non-targeting control pool siRNA (Horizon Discovery) using the normal human dermal fibroblast Nucleofactor kit (Lonza), following the manufacturer’s instruction and as previously described [22].

### Western blot

For Western blot analysis, protein concentrations were measured using the Micro BCA™ Protein Assay Kit (Thermo Fisher Scientific), 25 µg of protein was loaded onto SDS-PAGE gel, and the proteins were transferred to the PVDF membrane. Detecting antibodies included anti-CDH6 (48111), anti-ERK (4695), anti-phospho ERK (Thr202/Tyr204) (4370), anti-AKT (9272), anti-phospho AKT (Ser 473) (4060), anti-LC3 (3868), anti-GAPDH (2118) (all from Cell Signaling Technology). Horseradish peroxidase (HRP)-conjugated goat anti-rabbit IgG or anti-mouse IgG (Cell Signaling Technology, 7074 and 7076) were used as a secondary antibody. Blots were developed using Clarity ECL Western Blotting Substrates (Biorad), captured using a VersaDoc imaging system, followed by Image J analysis. The protein expression level was normalized to GAPDH.

### Immunofluorescence

Culture FLS were plated in chamber slides (Nalge Nunc International, Naperville, IL). After 3 days , cells were fixed with 4% paraformaldehyde for 15 min. After blocking with Bovine Serum Albumin (BSA) for 1 h in phosphate-buffered saline (PBS) and Tween 20 detergent. Specimens were incubated with affinity purified polyclonal rabbit anti-CDH6 IgG antibody (1:250) produced by Dr. Dressler, University of Michigan [23], or negative controls (no primary antibody or polyclonal rabbit IgG control (Abcam, ab37415)) for 2 h at room temperature. The slides were then washed and incubated with anti-rabbit IgG Alexa Fluor 594 (Invitrogen, A-11012). Nuclei were stained with 2ug/ml of 4’,6-diamidino-2-phenylindole (DAPI) (Invitrogen, D1306). Representative micrographs (20x) using Cytation 5 microscope and the confocal microscope Leica SP8 (40x and, 60x oil immersion objective).

For confocal microscopy, cryosections with 5 μm of synovial tissue were fixed in 4% paraformaldehyde for 15 min. The sections were blocked with BSA with PBSt for 1 h. Fc receptor block (Innovex) was added for 30 min. Followed by 2 h incubation with primary rabbit anti-CDH6 antibody (1:250) (from Dr. Dressler) and mouse anti-CD68 (1:50) (Santa Cruz, sc-20060), or with negative controls (no primary antibody, polyclonal rabbit IgG control, and mouse IgG control (Abcam, ab170190)). After washing, anti-Rabbit IgG Alexa Fluor 594 and anti-Mouse IgG Alexa Fluor 488 (Invitrogen, A-11012, and A-11059, respectively) were added for 1 h incubation. 2ug/ml of DAPI was used to stain the nucleus. Samples were imaged by the UCSD Cancer Center Core and representative micrographs (20x) were obtained using Olympus VS200 slide scanner.

### MTT cell growth assay

RA and OA FLS cell growth was assessed using MTT assays as previously described [24]. After siRNAs transfection, cells (3 × 10^3^ FLS/well in 96-well plates) were stimulated for 4 to 7 days with PDGF-BB (10 ng/ml). Cell growth was determined by incubation with MTT for 4 h. The plates were read at 550 nm with a spectrometer.

### Migration assay

After siRNAs transfection, RA FLS and OA FLS were plated (1.5 × 10^5^ FLS/well in 12-well plate), and after the serum starvation for 24 h, a linear scratch was created using a 1 ml micropipette tip as previously described [24]. Cells were incubated with or without PDGF-BB (10 ng/ml). Light microscopy images for four locations of marked wound were obtained at 0 and 24 h after wounding. The ECLIPSE E800 microscope (Nikon) with 40× magnification was used to obtain images. Migrated cells were counted by using ImageJ software.

### Cell cycle analysis

RA FLS were transfected with siRNA and cells were treated with PDGF-BB (10ng/ml) for 12 h, 24 h, 36 h, and 48 h. Cells were washed and fixed in 70% ethanol, and stained with propidium iodide supplemented with RNase A, for 30 min. The cells were analyzed by flow cytometry using a ZE5 Cell Analyzer (Biorad). Data were analyzed using FlowJo software (Tree Star), and percentages of cells in the G1/G0, S, and G2/M phases were calculated.

### Apoptosis assay

After siRNA transfection, RA FLS (4 × 10^3^ cell/well) were seeded in triplicates in 96-well plates after transfection, followed by serum starvation and then PDGF-BB (10ng/ml) treatment until day 3, followed by incubation for 15 h using the Apo-ONE^®^ Homogeneous Caspase-3/7 Assay kit. The assay was performed in accordance with the manufacturer’s instructions [22, 25]. Fluorescence was measured at a wavelength of 485 nm with emission maximum at 528 nm.

### Statistical analysis

Data are expressed as mean ± SEM, and it was used in Prism 10 (Graphpad). Statistics were performed with Student’s t-test, one-way analysis of variance, and repeated-measures analysis of variance as indicated. A comparison was considered significant at *p* < 0.05.

## Results

### CDH6 expression in RA FLS

**Fig. 1:**
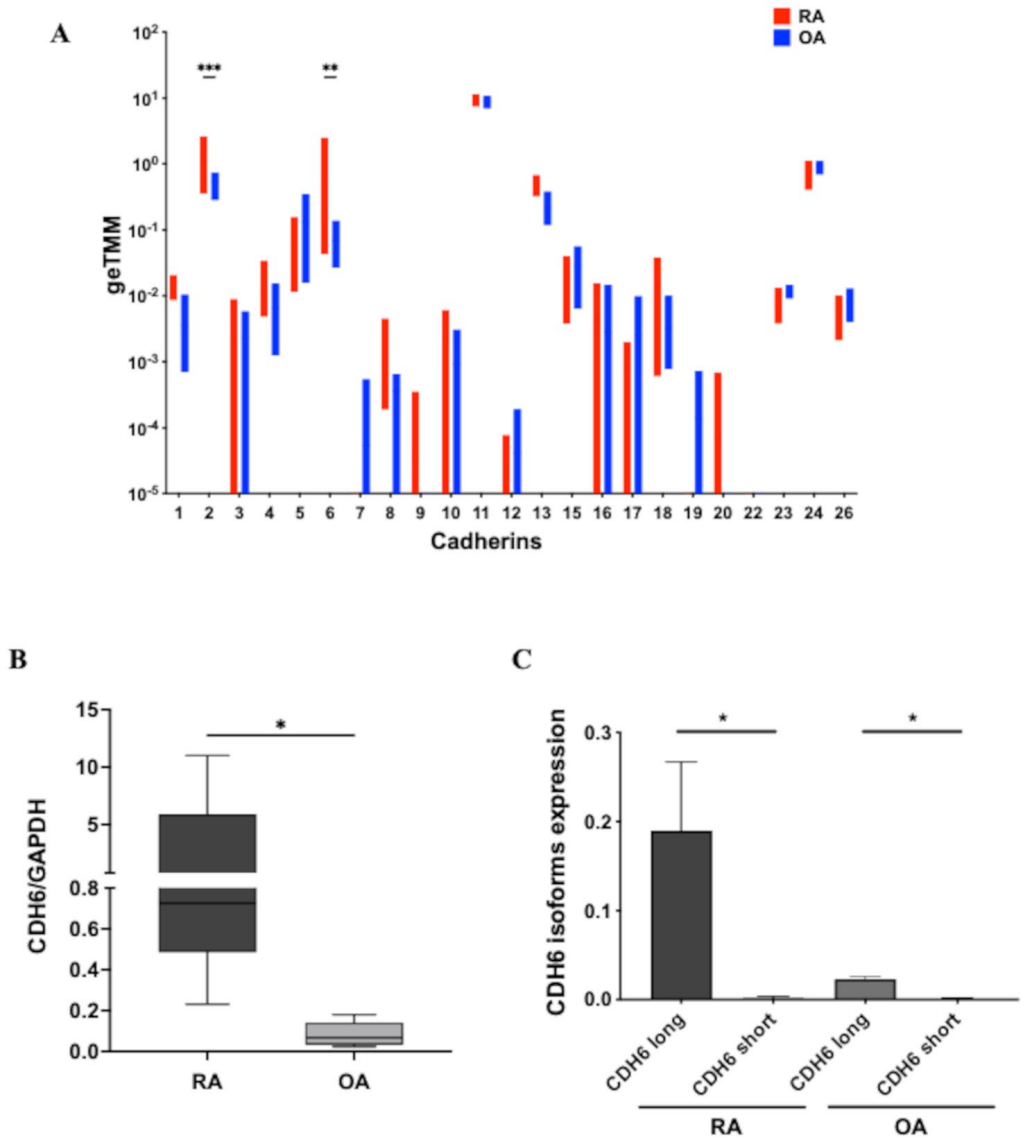
Expression of CDH6 in RA FLS and OA FLS. (**A**) Cadherin expression in RA FLS (red) and OA FLS (blue) as determined by RNAseq. CDH2 and CDH6 were significantly different for RA FLS vs. OA FLS (*n* = 10, each). (**B**) CDH6 mRNA expression measured by qRT-PCR in RA FLS and OA FLS (*n* = 6 each). CDH6 transcripts are higher in RA FLS than in OA FLS. (**C**) RNAseq data of CDH6 isoforms expression in RA FLS and OA FLS. The long form of CDH6 is highly expressed in FLS. All data were presented as mean ± SEM. *p ≤ 0.05 by Mann-Whitney test (**A**, **C**) and unpaired t-test (**B**)

We began our evaluation of OA and RA FLS cadherin expression by analyzing RNAseq data to quantify mRNA transcripts of cadherin family members [20]. Figure 1A shows that most of the cadherins are expressed by cultured FLS, and we noted that only CDH2 (also called N-cadherin) and CDH6 were differentially expressed when comparing OA and RA FLS (*p* < 0.01). Of interest, CDH11 was equally expressed in OA and RA FLS. We were particularly interested in CDH6 because of its role in cancer and its potential contribution to aggressive behavior displayed by RA FLS [4, 5]. To confirm the RNAseq data showing higher expression in RA than OA FLS, we used RT-qPCR on additional cell lines. As expected, greater levels were detected in RA FLS (*p* < 0.05) (Fig. 1B). We also defined the splice forms expressed in FLS. CDH6 has two splicing variants, the canonical isoform 1 (long form), which comprises an extracellular and cytoplasmic domain, and the isoform 2 (short form), which presents an extracellular domain but lacks the cytoplasmic domain [26]. Figure 1C shows that the long form is the primary transcript in FLS (*p* < 0.005).

### CDH6 epigenetic profile and mechanism

**Fig. 2:**
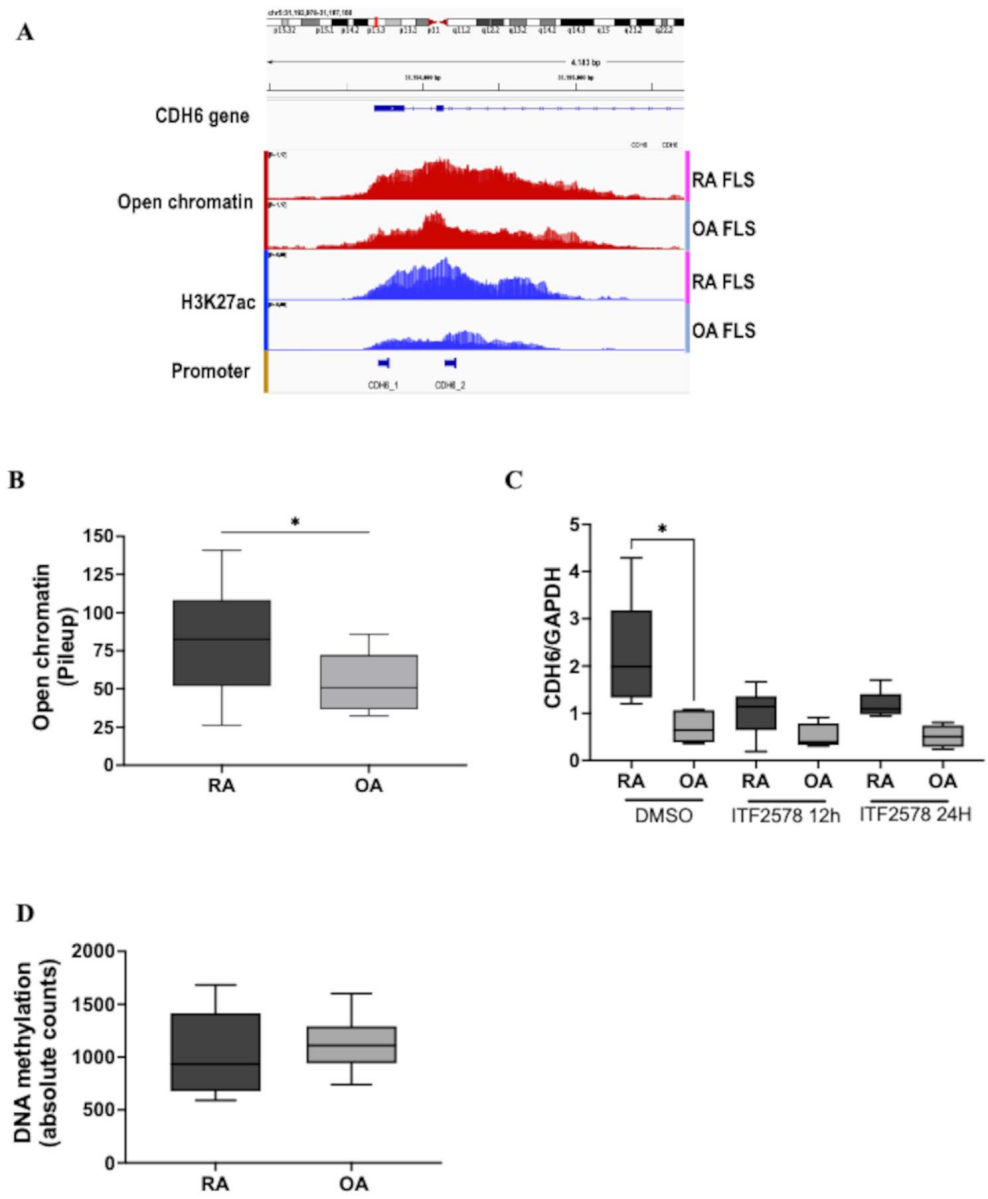
Epigenetic profile of the CDH6 promoter region in RA FLS and OA FLS. (**A**) Genome browser view of open chromatin, H3K27ac modifications in the CDH6 promotor region. (**B**) Pileup visualization of open chromatin (OA: *n* = 11, RA: *n* = 11) calculated by area under curves (AUC). (**C**) RA and OA FLS were incubated with 1µM of ITF2357 (pan HDAC inhibitor) for 12 h and 24 h, and CDH6 mRNA levels were measured by qRT-PCR (*n* = 5). CDH6 was reduced after ITF2357 treatment in RA FLS (OA: *n* = 5, RA: *n* = 5). (**D**) Absolute counts of DNA methylation in CDH6 promoter region in RA FLS and OA FLS, no significant differences were found. All data were presented as mean ± SEM. * *p* ≤ 0.05 by unpaired t-test (**B** and **D**) and Kurskal-Wallis test (**C**)

To understand the mechanism of greater CDH6 expression in RA compared with OA FLS, we used our previously published ATACseq, ChIPseq, and DNA methylation data to examine the chromatin accessibility, histone modifications, and DNA methylation of CDH6 regulatory regions in RA FLS and OA FLS [20]. As shown in Fig. 2A and B, chromatin accessibility was significantly greater in the promoter region of RA versus OA FLS (1.45-fold RA/OA, *p* = 0.03), which is consistent with increased transcriptional activity in RA FLS. There was a trend toward higher H3K27ac peaks in RA FLS (2.25-fold RA/OA) (*p* = 0.0557), which correlates with chromatin accessibility when compared to OA FLS (Fig. 2A). We then validated these results by treating FLS with ITF2357, a pan histone deacetylase (HDAC) inhibitor [19]. The difference in CDH6 expression between RA and OA FLS was eliminated by the HDAC inhibitor (Fig. 2C). These findings suggest that H3K27ac, and most likely resultant chromatin accessibility, contributes to the mechanism of higher CDH6 expression in RA FLS. We then examined the DNA methylation in the CDH6 promoter [20], but no differences were found between RA and OA FLS (Fig. 2D). Therefore, we concluded that differential CDH6 expression in RA is predominantly regulated by histone modifications rather than DNA methylation.

### CDH6 protein and distribution in FLS

**Fig. 3:**
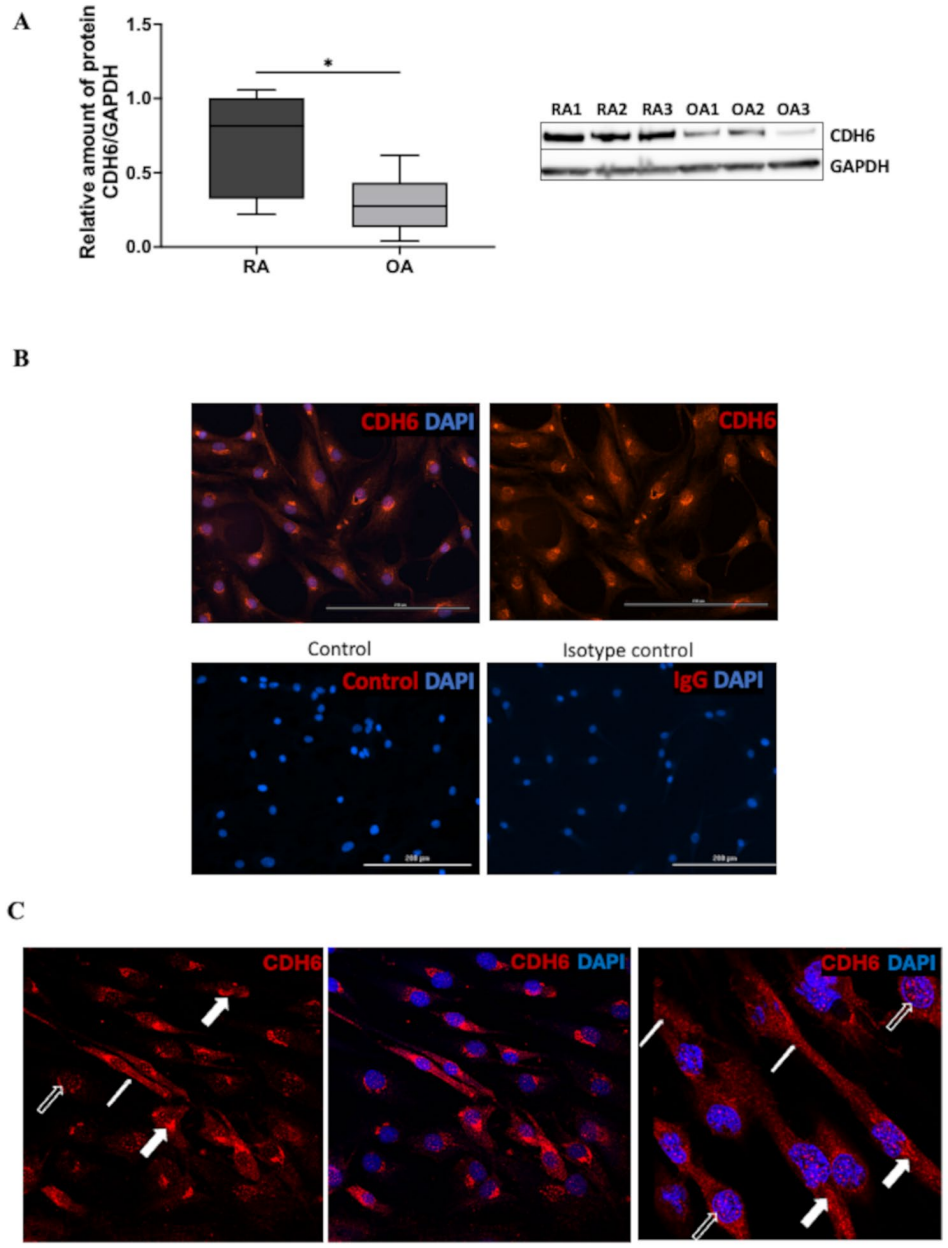
CDH6 protein expression and distribution in FLS. (**A**) CDH6 protein level of CDH6 in RA FLS and OA FLS (*n* = 7 each). Representative western blot images of CDH6 and GAPDH from RA FLS and OA FLS are shown. As with gene expression, CDH6 protein is greater in RA FLS than OA FLS. (**B**) Using immunofluorescence, CDH6 protein was identified in the nucleus, perinucleus, cytoplasm, and membrane in RA FLS. RA FLS were cultured in chamber slides and stained with CDH6 antibody, followed by Alexa Fluor 594. As negative controls, no primary antibody and polyclonal rabbit IgG at the same concentration as the CDH6 antibody were used (bottom left and right, respectively), followed by Alexa Fluor 594-conjugated secondary antibody. Representative images using fluorescence microscopy Cytation 5. (**C**) Distribution of CDH6 in RA FLS confirmed by confocal microscopy. Representative images were captured by confocal microscopy using a Leica SP8 microscope (40x and 60x). CDH6 expression in the nucleus (open arrow), in the perinuclear region (thick arrow), and cytoplasm and membrane (thin arrow). All data were presented as mean ± SEM. *P ≤ 0.05 by unpaired t-test

To confirm the relevance of the promoter region and mRNA levels, we examined CDH6 protein expression by Western Blot. As shown in Fig. 3A, CDH6 protein levels were higher in RA compared to OA FLS. We then explored the protein localization in RA FLS by immunostaining. Some cadherin proteins, most notably E-cadherin (ECDH), localize in the cell nucleus, where they can potentially participate in intracellular processes [27, 28]. Using fluorescence and confocal microscopy, CDH6 was detected in the cell membrane and cytoplasm. However, the protein also localized to the nucleus and peri-nuclear regions in FLS (Fig. 3B and C). This raises the possibility that some CDH6 functions, as with E-cadherin, could be the result of intracellular localization.

### CDH6 regulation

**Fig. 4:**
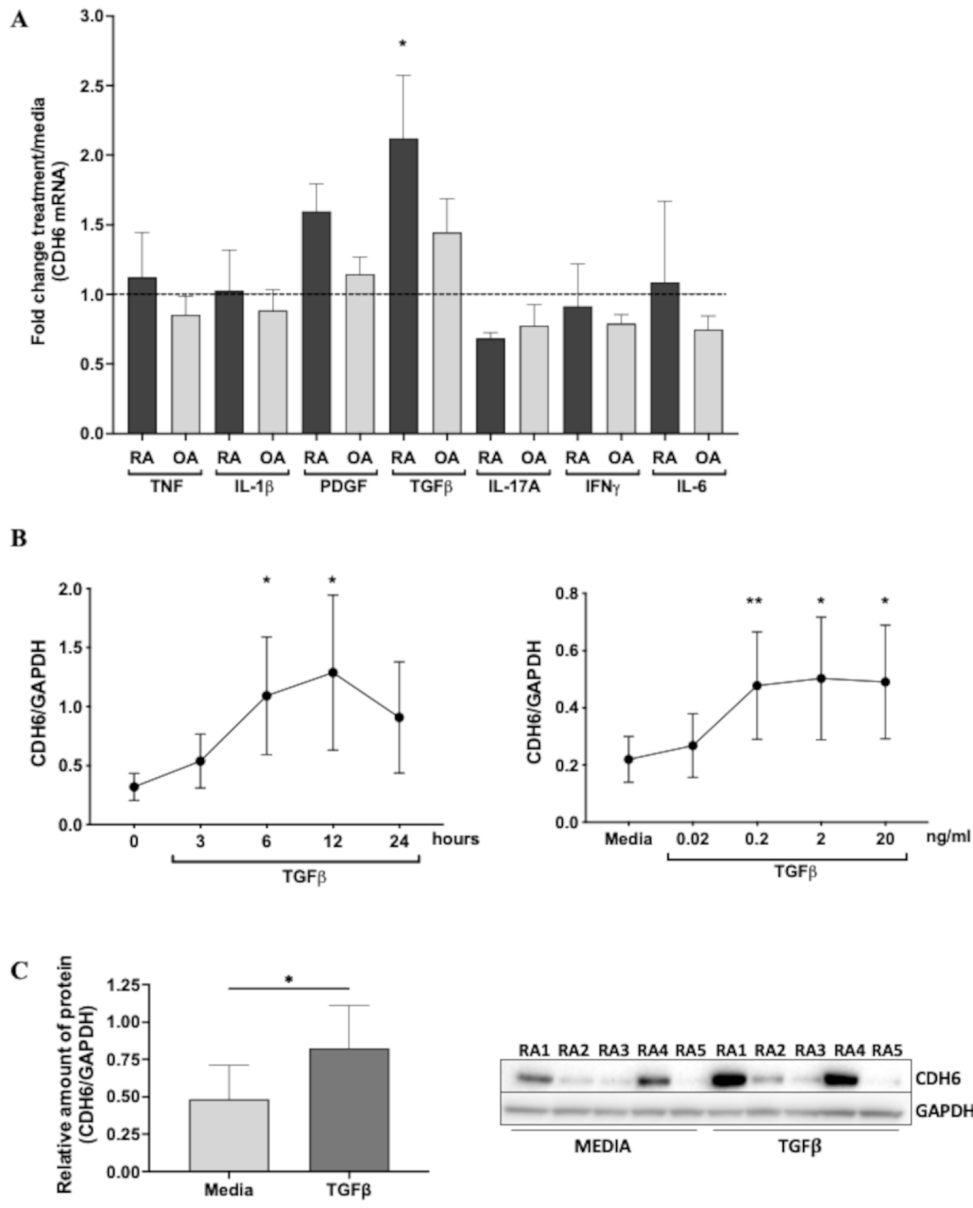
Regulation of CDH6 in FLS. (**A**) CDH6 was measured in OA and RA FLS by qRT-PCR after stimulation with TNF (50 ng/ml), IL-1β (2ng/ml), PDGF (10ng/ml), TGFβ (20ng/ml), IL-17A (50ng/ml), IFNγ (10ng/ml), or IL-6 (20ng/ml) for 6 h. The graph shows fold change value compared with RA and FLS cultured in medium alone (*n* = 5–7 each). (**B**) Dose-response and kinetics of CDH6 induction by TGFß. Left side: mRNA expression of CDH6 in RA FLS stimulated with TGFβ (0, 0.02, 0.2, 2, or 20 ng/ml for 6 h) (*n* = 6). Right side: Time course of mRNA expression of CDH6 in RA FLS stimulated with 20 ng/ml TGFβ (0, 3, 6, 12, or 24 h) (*n* = 6). (**C**) CDH6 protein expression in RA FLS stimulated with TGFß (20 ng/ml for 24 h) (*n* = 4). All data were presented as mean ± SEM. Analysis was performed using the Friedman test (**A** and **B**) and Wilcoxon test (**C**). * *p* ≤ 0.05

To evaluate CDH6 regulation, we determined gene expression after exposure to cytokines and growth factors that reflect the inflammatory environment in RA joints. RA FLS and OA FLS were stimulated with TNF, IL-1β, PDGF, TGFβ, IL-17A, IFNγ, or IL-6, and CDH6 mRNA was measured by q PCR (Fig. 4A). TNF, IL-1β, PDGF, IL-17A, IFNγ, or IL-6 stimulation did not significantly affect CDH6 expression in either RA or OA FLS. However, CDH6 expression was significantly increased by TGFß in RA FLS (2.12-fold, *p* = 0.03) but with only a trend toward an increase OA FLS. We then performed the dose-response and kinetics studies of TGFβ-mediated induction (Fig. 4B). Maximum expression was observed at 0.2 ng/ml, and CDH6 transcripts peaked after 12 h of stimulation (20 ng/ml) (*p* < 0.05). CDH6 protein levels in individual RA FLS lines were highly variable, but an increase was observed after 24 h of TGFß stimulation (Fig. 4C).

### CDH6 regulation of OA and RA FLS cell growth

**Fig. 5:**
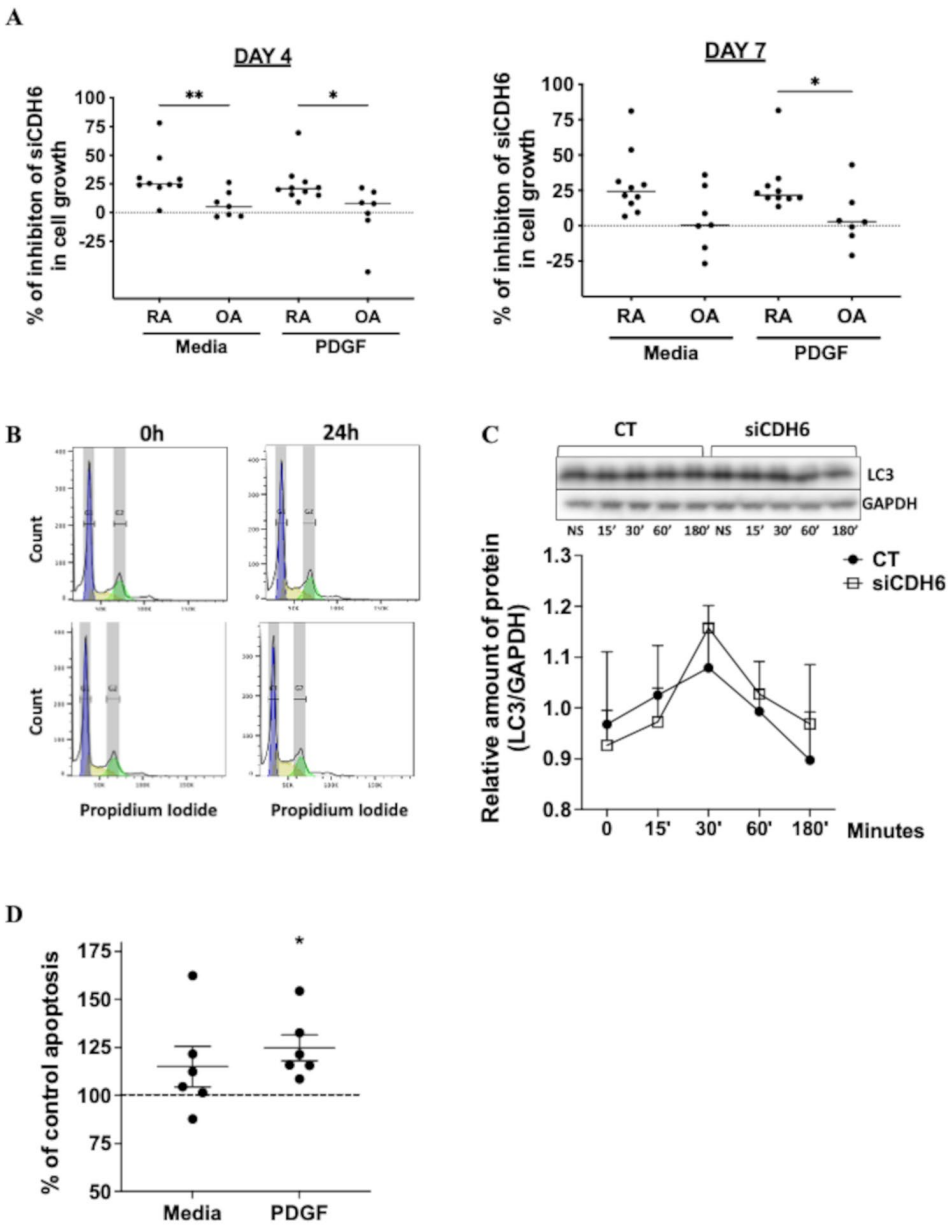
CDH6 regulates RA FLS cell survival. (**A**) Effect of CDH6 knockdown on FLS growth. OA and RA FLS were transfected with siCDH6 or CT and cultured in the presence of PDGF (10 ng/mL) for 4 and 7 days in triplicate wells. Data shows the percentage of inhibition after knockdown CDH6 in cell growth in RA FLS (*n* = 10) and OA FLS (*n* = 7), RA FLS is significantly higher than OA FLS. Graphs represent the percentage of inhibition of cell growth induced by siCDH6 compared to CT in both RA and OA FLS. (**B**) Cell cycle analysis after CDH6 knockdown and stimulated by PDGF. RA FLS were transfected with siRNA, serum-starved, and induced with 10ng/ml of PDGF. Representative histograms of DNA content in RA FLS lines transfected with CT and siCDH6 in 0 h and 24 h (*n* = 3). CDH6 had no effect on any phase of the cell cycle. (**C**) Autophagy analysis after CDH6 knockdown. FLS were cultured with PDGF (10 ng/mL) for 15, 30, 60, and 180 min with or without knockdown. The autophagy marker LC3 and GAPDH protein were measured by Western blot analysis. Representative images of LC3 cultured with or without PDGF in CT and siCDH6 transfected cells are shown (*n* = 4). (**D**) Effect of CDH6 knockdown on FLS apoptosis. FLS transfected with siCDH6 and cultured with or without PDGF (10 ng/ml), then incubated for 15 h using the Apo-ONE^®^ Homogeneous Caspase-3/7 Assay kit (*n* = 6). Data is shown as the percentage of the ratio between siCDH6 and the control, showing apoptosis is higher after knockdown. All data were presented as mean ± SEM. *P ≤ 0.05, Kruskal-Wallis multiple comparison test (**A**) and ANOVA multiple comparison test (**C** and **D**)

CDH6 has been implicated in tumor progression and cancer metastasis, potentially through its effects on migration, autophagy, and cell growth [5]. To determine the role of CDH6 in these biological functions, CDH6 was reduced by over 85% using siRNA at 24 h, 48 h, and 72 h (Fig. S1A and B). Cells without stimulation (Media) and PDGF-induced cell growth were assessed using an MTT assay. As expected, PDGF-induced increased cell growth in control cells (Fig S2A and B). In RA FLS, CDH6 knockdown significantly decreased cell growth on days 4 and 7 in media and PDGF-induced conditions (Fig. S2A and B). Figure 5A represents the percentage of inhibition of cell growth induced by siCDH6 compared to CT in both RA and OA FLS. The inhibitory effect of siCDH6 was higher in RA FLS than in OA FLS in media and PDGF-induced cells. For example, CDH6 knockdown reduced cell growth in RA FLS by 30% and 25% in media and PDGF-induced conditions, respectively, on day 4, and by 27% under both conditions on day 7 (*p* < 0.05). In contrast, no significant reduction was observed in OA FLS, with − 0.4% and 7% on day 4 and 4% and 5% on day 7 in media and PDGF-induced conditions, respectively. To confirm if the cell growth effect in RA FLS is due to CDH6 surface expression, we used an anti-CDH6 antibody construct derived from the extracellular domain of the human cadherin-6 fusion protein in non-knockdown RA FLS. However, no differences were detected using the antibody, suggesting that other intracellular processes might regulate this function (Fig. S2C).

To understand the mechanism by which CDH6 affects cell growth, we evaluated pathways associated with these processes, such as phospho-ERK and phospho-AKT. No differences were detected when comparing control with siCDH6 FLS (Fig. S3B). We then examined whether CDH6 knockdown affects the cell cycle by flow cytometry, but no effect was observed (Fig. 5B and S3A). Given the lack of effect on cell division, we then evaluated whether changes in cell survival could explain the cell growth results. CDH6 has been implicated in autophagy [11], so we determined whether knockdown affects this process by measuring LC3, an autophagy marker. However, no differences were observed, indicating that altered autophagy is not responsible for cell growth effects (Fig. 5C). As a positive control for this experiment, we tested chloroquine, an autophagy inhibitor that increases LC3 protein levels, was unaffected by CDH6 knockdown (Fig. S3C). Given the lack of evidence for a direct effect on cell proliferation, we evaluated whether decreased cell survival through apoptosis might contribute. Therefore, we measured caspase 3 and 7 activity after CDH6 knockdown in RA FLS in media and PDGF-induced cells, as these enzymes are specific markers for apoptosis. Figure 5D shows that CDH6 knockdown increased caspase 3 and 7 (*p* < 0.02), the data is shown as a percentage of CDH6 knockdown relative to control. These data suggest that the effect of CDH6 increased cell growth by reducing apoptosis in RA FLS cells.

### CDH6 regulation FLS migration

**Fig. 6:**
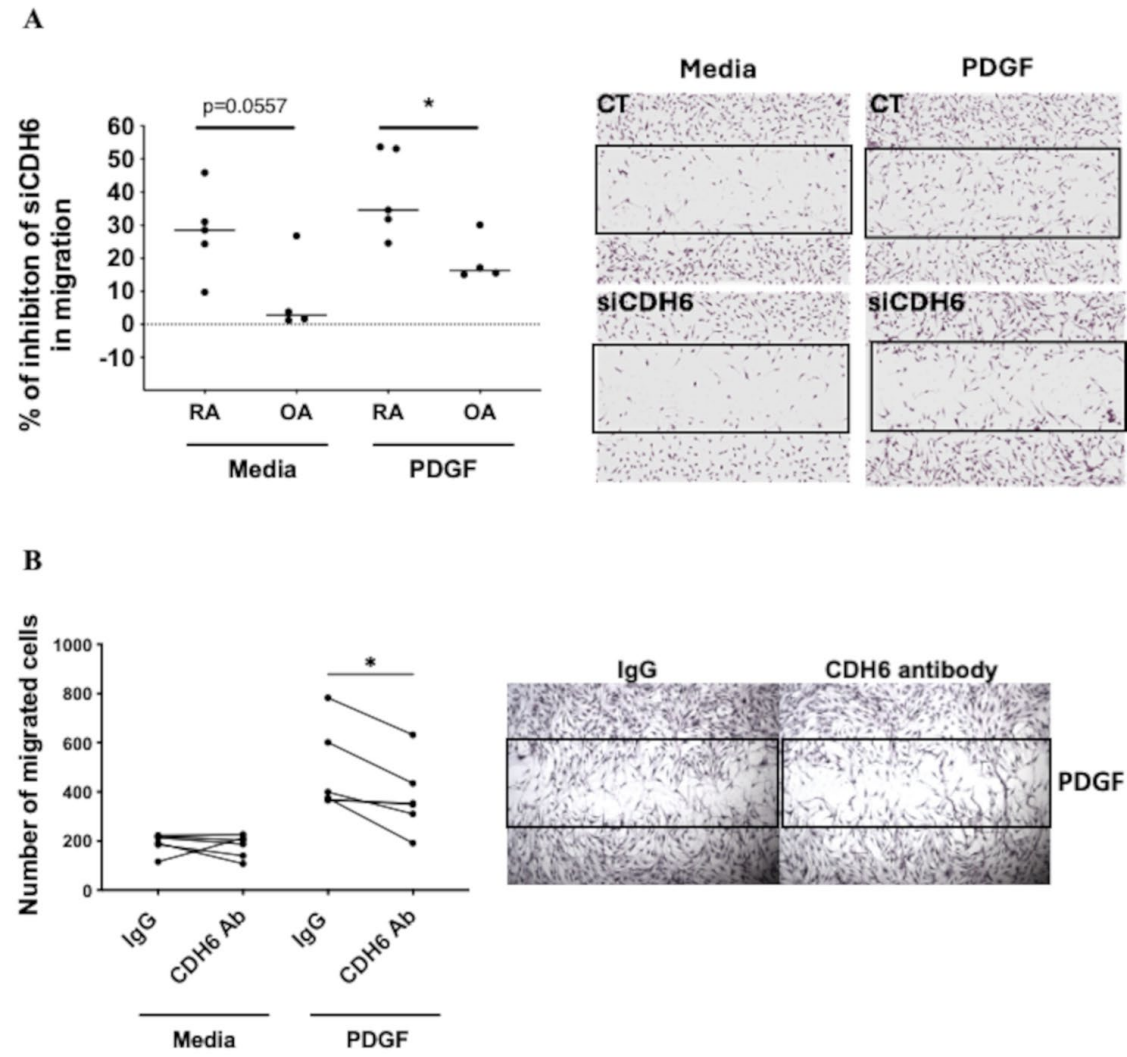
CDH6 regulates RA FLS migration. (**A**) Effect of CDH6 knockdown on FLS migration. Right side: representative wound healing assay of RA FLS transfected with CDH6 siRNA (siCDH6) or scramble control (CT) with or without PDGF-stimulation (10 ng/ml for 24 h). Left side: Graphs represent the percentage of migration inhibition induced by siCDH6 compared to CT in both RA and OA FLS (RA: *n* = 6 and OA: *n* = 4). (**B**) Effect of Anti-CDH6 on RA FLS migration. Right side: representative migration assay with or without PDGF stimulation in RA FLS (10 ng/ml for 24 h). Cultures included 2ug/ml of anti-CDH6 antibody or anti-IgG antibody. Left side: Quantification of cells that migrate into the wound after 24 h of PDGF stimulation (*n* = 6). All data were presented as mean ± SEM. *P ≤ 0.05, ANOVA multiple comparison test

CDH6 has also been shown to have a role in cancer by affecting cell migration [5, 14]. As observed for cell growth, PDGF stimulation also promoted FLS migration compared to unstimulated cells (Fig. S2D and E). Knockdown of CDH6 significantly reduced migration in RA FLS and OA FLS in media and PDGF-induced cells (*p* < 0.05) (Fig. S2D and S2E). When comparing the percentage of inhibition, CDH6 knockdown showed greater inhibition of migration in RA FLS than in OA FLS (*p* < 0.05) (Fig. 6A). For example, in PDGF-induced migration, the CDH6 knockdown showed 34% inhibition for RA FLS and 19% inhibition for OA FLS,. Furthermore, CDH6 knockdown inhibited migration similarly between media and PDGF. To verify whether this effect involves CDH6 surface expression, we also used an anti-CDH6 antibody in non-knockdown FLS. Figure 6B shows that the antibody significantly decreased the migration of PDGF-induced cells (*p* < 0.03).

To investigate the mechanism of CDH6 in migration, we also evaluated pathways associated with this process, including RAC1 and PAK1 (data not shown), as well as phospho-ERK and phospho-AKT (Fig. S3B). No differences were observed between the siCDH6 and the control FLS, indicating that CDH6 knockdown likely disrupts downstream pathways.

### CDH6 distribution in synovial tissue

**Fig. 7:**
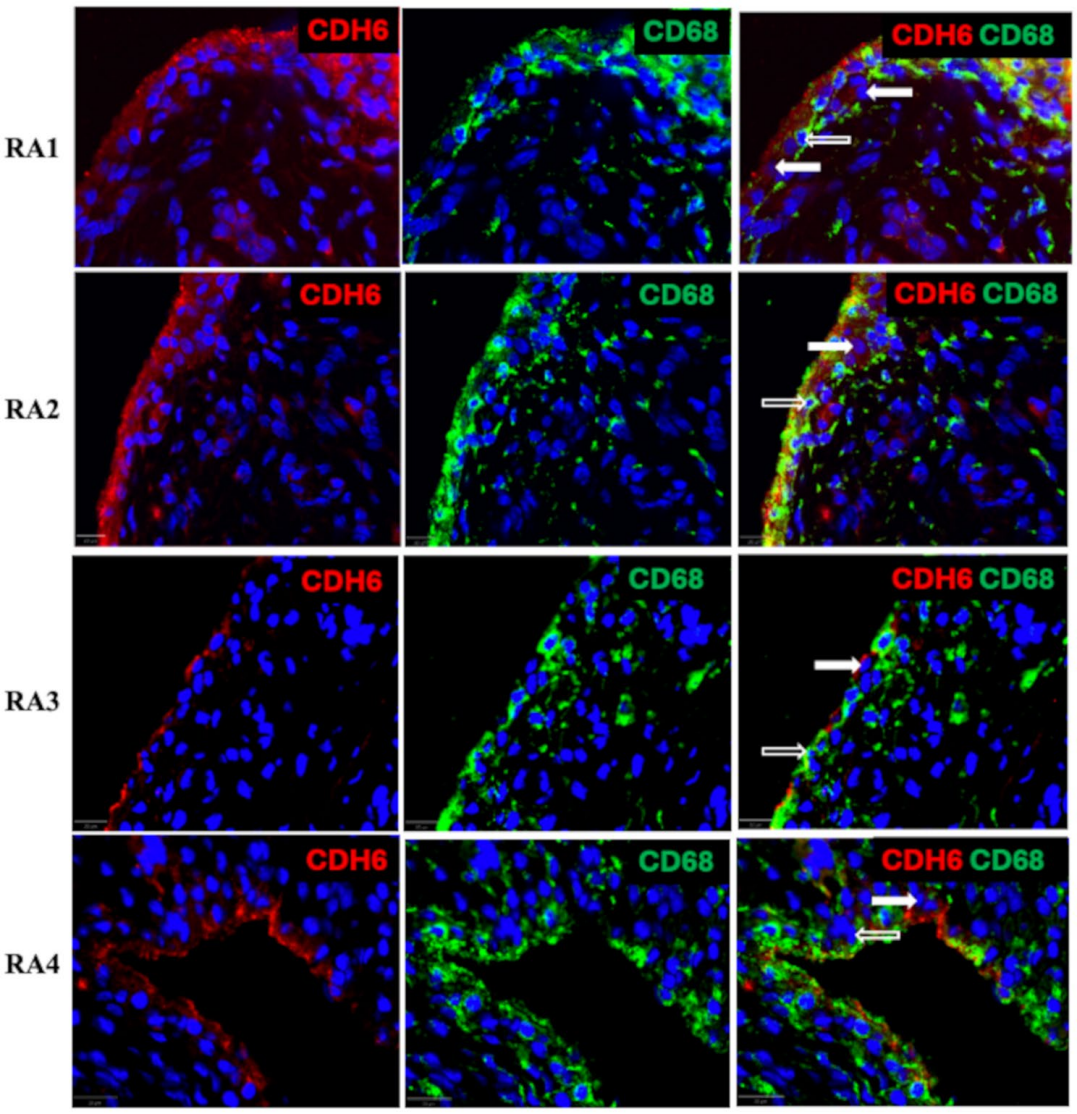
Distribution of CDH6 protein in RA synovial tissue using confocal microscopy. Representative images of RA synovium stained with anti-CDH6 and -CD68 (macrophage) antibodies demonstrate the lining and sublining expression of CDH6 (type A and B synoviocytes), most prominently in lining FLS (solid white arrow) and macrophages (open arrows) (*n* = 4 separate tissues were stained). CDH6 and CD68 are also expressed in the sublining region. Images were taken with Olympus VS200 slide scanner (20x) high resolution. S4 Figure shows the negative controls for these images (no primary antibody or control rabbit IgG, and control mouse IgG)

FLS generally reside in the synovial intimal lining, although multiple fibroblast phenotypes can be found in sublining regions [3, 4]. Because cultured RA FLS highly expressed CDH6, we evaluated whether the protein is also found in the rheumatoid synovial lining fibroblasts using high-resolution confocal microscopy in tissues stained for CD68 and CDH6. Anti-CD68 stains macrophage-like synoviocytes (MLS) that are located in intimal lining regions, as well as other macrophages located in sublining regions. Figure 7 shows CDH6 protein expression in lining CD68 + macrophages and CD68- cells (non-macrophages) and confirms the abundant expression of this cadherin molecule in this region in both CD68 positive and negative cells. CDH6 was expressed to a lesser degree in the sublining cells, particularly macrophages, and non-macrophages in perivascular locations.

## Discussion

Cadherins can play a crucial role in fibroblast function by regulating cell-cell adhesion and maintaining tissue architecture. They can serve as signaling proteins and are involved in cellular junctions, migration, and maintaining tissue homeostasis [8, 9, 29]. In RA, CDH11, in particular, has been implicated as a key protein required for FLS homotypic aggregation in the synovial intimal lining [7, 8]. To understand if there are broader functions of the cadherin family in synovial biology, we evaluated the expression of other cadherins in FLS. Unexpectedly, we identified another cadherin, namely CDH6, as a gene of interest in RA. Notably, CDH6 is expressed at higher levels in RA FLS compared with OA FLS and promotes an aggressive phenotype, potentially through a mechanism involving migration and cell survival. These studies suggest that targeting CDH6 in RA might have potential clinical benefit by attenuating the aggressive behaviors of FLS. This molecule does not appear to play a major role in immune responses, possibly enabling its use in combination with agents that affect host defense. In addition, this approach could mitigate fibroblast-mediated structural damage when it progresses despite suppressed inflammation or could be used as a biomarker to identify patients with fibroblast activation.

CDH6 is a type II cadherin and is highly expressed in kidneys, brain, and liver. It is overexpressed in a variety of tumors, including renal carcinomas, gliomas, thyroid cancer, gastric cancer, and breast cancer [5, 10–12, 30, 31]. Its expression also correlates with lymph node invasion and metastasis, serving as a marker for a poor prognosis [32]. Our study shows for the first time that CDH6 expression is also elevated in RA FLS, which display many features of aggressive tumor cells, such as enhanced invasiveness and survival [5, 26, 31, 33]. In synovial tissue, its expression is observed mainly in FLS, but it is also displayed by MLS in intimal lining regions, as well as other cell types in sublining regions. The interaction between FLS and MLS through adhesion molecules was seen previously, such as the VCAM-1 and integrin α4β1, which are known to contribute to the structure of the synovium in vivo [7]. Interestingly, it was observed that CDH6 is associated with immune infiltration and cytokine expression in glioma, suggesting that the CDH6 presence may facilitate the interaction between FLS and MLS, potentially contributing to the hyperplastic lining and chronic inflammation.

CDH6 has two splice variants: a long form that includes an extracellular domain with a cytoplasmic domain and a short form lacking this domain [26]. Of interest, certain cadherins have a signaling role by interaction with downstream molecules through their cytoplasmic domains, such as E-CDH and N-CDH [27, 28, 34]. In thyroid tumor cells, both CDH6 isoforms are expressed [26, 31]. However, in FLS the long form is much more abundant, suggesting that intracellular interactions of CDH6 might contribute to the function of CDH6. Interestingly, we also found abundant CDH6 intracellular distribution in FLS, including localization to the nucleus and perinuclear regions, further suggesting CDH6 might contribute to intracellular signaling mechanisms. The intracellular distribution could also explain why the CDH6 antibody did not block cell growth compared with siRNA knockdown in FLS in our studies. In contrast, the CDH6 antibody did inhibit migration, indicating that both the extra- and intra-cellular domains of CDH6 play roles in FLS biology.

Increased expression of CDH6 in RA FLS is associated with specific epigenetic marks associated with other pathologies. For example, a high level of tri-methylation on lysine 27 of histone H3 (H3K27me3) at the CDH6 gene locus is observed in multiple myeloma cells, and this modification correlates with low CDH6 expression [35]. To help understand increased expression in RA, we reviewed epigenetic landscape data and discovered that chromatin accessibility and histone modifications, particularly H3K27ac, are enhanced in RA FLS compared with OA FLS. H3K27Ac is closely associated with open chromatin and gene expression, which likely explains the higher CDH6 transcription in RA FLS. To confirm this, we treated FLS with a histone deacetylase inhibitor, which equalized CDH6 expression between RA and OA FLS. These data suggest that histone acetylation plays a critical role in the differential regulation of CDH6 in RA.

In addition to epigenetic markers, we also showed that CDH6 is induced by TGFβ in RA FLS. Previous studies have shown that CDH6 expression is increased by TGFβ during epithelial-to-mesenchymal transition in cancer, promoting invasiveness in tumor cells [10, 26]. TGFβ is a multifunctional cytokine involved in diverse biological responses, such as angiogenesis, cell proliferation, differentiation, migration, and apoptosis, and it plays a pivotal role in fibrosis and pathogenesis associated with RA [36, 37]. Our data suggests that CDH6 may act as a downstream mediator of TGFβ signaling in the RA synovium. However, further investigation is necessary to understand this mechanism.

CDH6 function has been implicated in cancer cell migration, invasion, and cell growth [5, 13, 33, 38]. For example, CDH6 knockdown decreases migration and invasion in ovarian and renal cancer cell lines, which was attributed to reduced AKT and ERK activation [31]. We showed that CDH6 regulates similar functions in RA FLS that might contribute to their aggressive behaviors. However, in RA FLS, mechanism studies did not implicate AKT, ERK, or cell cycle abnormalities. Surprisingly, autophagy in FLS was also not regulated by CDH6, even though that process has been implicated in other cell types [11]. However, we did demonstrate that CDH6 knockdown modestly decreases cell survival, offering an explanation for its effect on cell growth.

These studies have some limitations. First, our findings are based on in vitro experiments using primary FLS cultures, which might not reflect the situation in situ. Even though all FLS lines express CDH6, there is variability in CDH6 expression among FLS lines. Therefore, we used multiple lines to ensure that we had representative samples. Also, we were able to determine the effect of CDH6 on cell survival, but the precise molecular mechanism remains unknown. We suspect that it is related to intracellular functions of the long form. The role of CDH6 in joint development or structure is also an area that is not fully explored. Our studies focused on in vitro assays, and the effect of CDH6-directed approaches in pre-clinical arthritis models would be helpful. However, the interpretation of these experiments might be difficult because mouse CDH6 has distinct functions compared with the human protein. For example, CDH6 interacts with αIIb/ß3 on platelets and is associated with thrombosis in humans but operates through a different non-platelet mechanism in mice [39]. Finally, our studies focused on patients with longstanding disease requiring arthroplasty, although it would be interesting to evaluate early-stage disease to determine if outcomes correlate with CDH6 expression and function.

In conclusion, our findings reveal that CDH6 is differentially expressed in RA FLS over OA FLS and plays a significant role in RA FLS pathogenic behavior, sharing regulatory and functional similarities to its role in cancer. The increased differential RA FLS expression may contribute to FLS survival and migration in situ, contributing to RA pathology and chronicity. Disrupting CDH6-mediated cell interaction may reduce synovial hyperplasia in RA. CDH6 has been shown to be an attractive target for treating certain cancers. For example, a phase I study of HKT288, a CDH6-targeting antibody-cytotoxic drug conjugate, was terminated due to neurologic adverse events [15]. Another phase 1a dose-escalation has been reported with another antibody-payload approach [16]. Given CDH6 involvement in aggressive phenotypes in RA, these CDH6-targeted approaches might be useful in RA as well.

## Supplementary Information

Below is the link to the electronic supplementary material.

Supplementary Material 1

Supplementary Material 2

Supplementary Material 3

Supplementary Material 4

Supplementary Material 5

Supplementary Material 6

Supplementary Material 7

Supplementary Material 8

Supplementary Material 9

Supplementary Material 10

## Data Availability

No datasets were generated or analysed during the current study.

## Abbreviations

RA: Rheumatoid Arthritis
OA: Osteoarthritis
FLS: Fibroblast–like Synoviocytes
CDH6: Cadherin 6
CDH11: Cadherin 11
E-CDH: E–cadherin
N-CDH: N-cadherin
IL-1ß: Interleukin–1 Beta
TNF: Tumor Necrosis Factor
TGFβ: Transforming Growth Factor Beta
PDGF: Platelet–Derived Growth Factor
PBS: Phosphate-buffered Saline
H3K27ac: Histone 3 Lysine 27 Acetylation
H3K27me3: Histone 3 Lysine 27 Trimethylation
ERK: Extracellular Signal–Regulated Kinase
AKT: Protein Kinase B
JAK: Janus Kinase
RAC1: Ras–Related C3 Botulinum Toxin Substrate 1
PAK1: p21–Activated Kinase 1
LC3: Microtubule associated Protein 1 Light Chain 3 Alpha
GAPDH: Glyceraldehyde 3–phosphate Dehydrogenase
RT-PCR: Reverse Transcription Polymerase Chain Reaction
qPCR: Quantitative Polymerase Chain Reaction
RNAseq: RNA Sequencing
ATACseq: Assay for Transposase–Accessible Chromatin using Sequencing
ChIPseq: Chromatin Immunoprecipitation Sequencing
DMEM: Dulbecco’s Modified Eagle Medium
FCS: Fetal Calf Serum
siRNA: Small Interfering RNA
HDAC: Histone Deacetylase
ITF2357: Pan Histone Deacetylase Inhibitor
MTT assay: 3–(4,5–Dimethylthiazol–2–yl)–2,5–Diphenyltetrazolium Bromide Assay
DAPI: 4’,6–Diamidino–2–Phenylindole (Fluorescent Nuclear Stain)
ACR: American College of Rheumatology
GEO: Gene Expression Omnibus
