# Supplementary figures and images for: Regulation of fibroblast-like synoviocyte function by cadherin 6 in rheumatoid arthritis
Source: Arthritis Res Ther. 2025 Aug 29;27:172. doi: 10.1186/s13075-025-03637-1 (PMC12395867; doi:10.1186/s13075-025-03637-1)

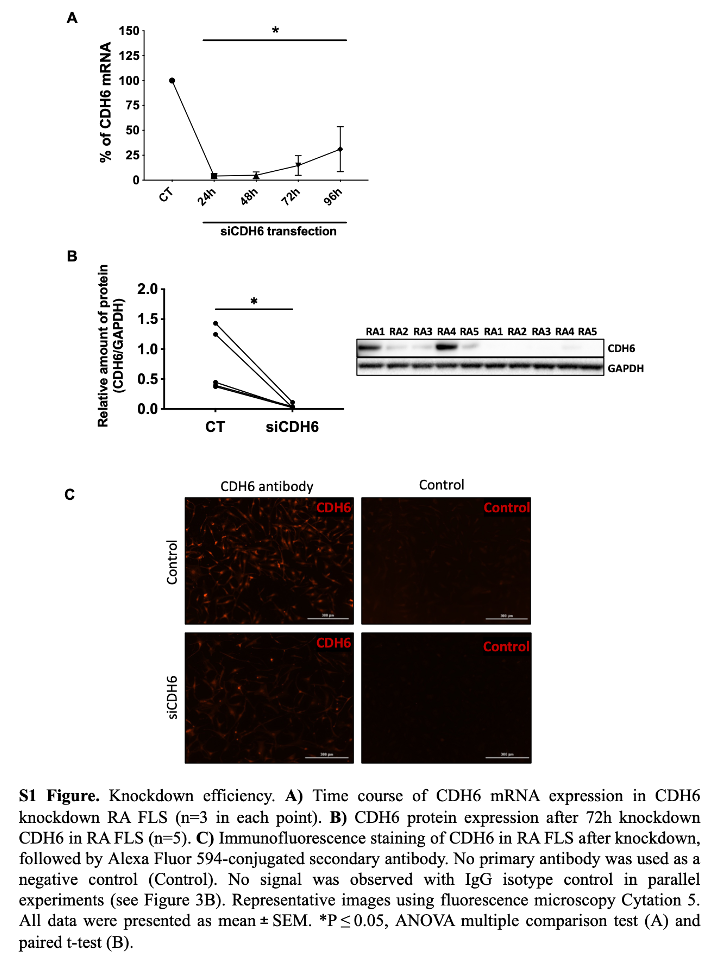

Supplement: Supplementary file 1 — Supplementary Material 1 [file 13075_2025_3637_MOESM1_ESM.tiff]

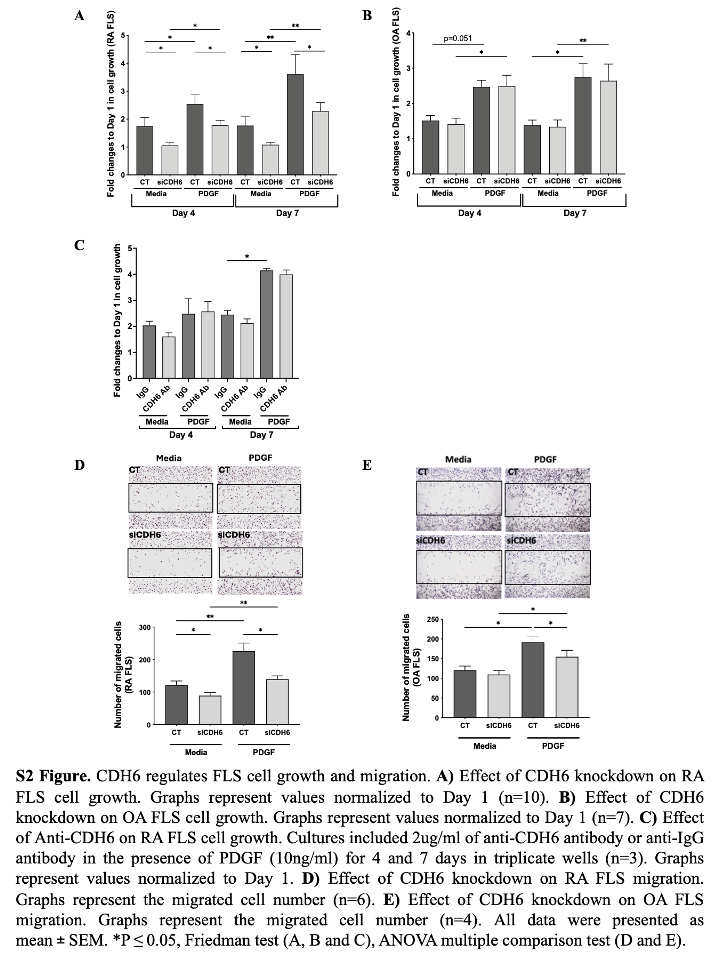

Supplement: Supplementary file 2 — Supplementary Material 2 [file 13075_2025_3637_MOESM2_ESM.tiff]

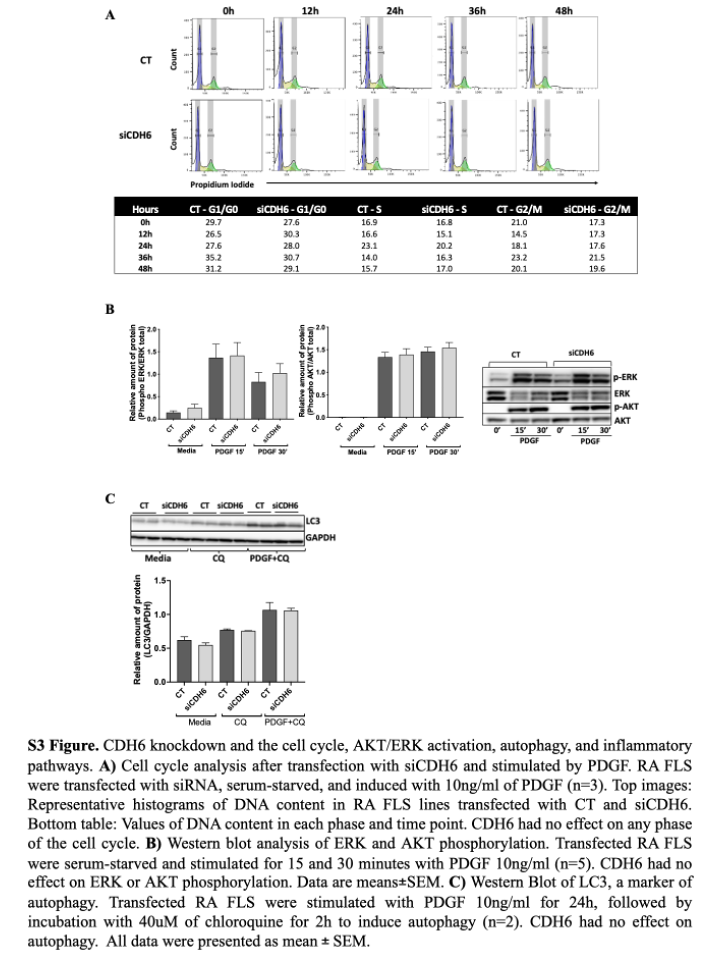

Supplement: Supplementary file 3 — Supplementary Material 3 [file 13075_2025_3637_MOESM3_ESM.tiff]

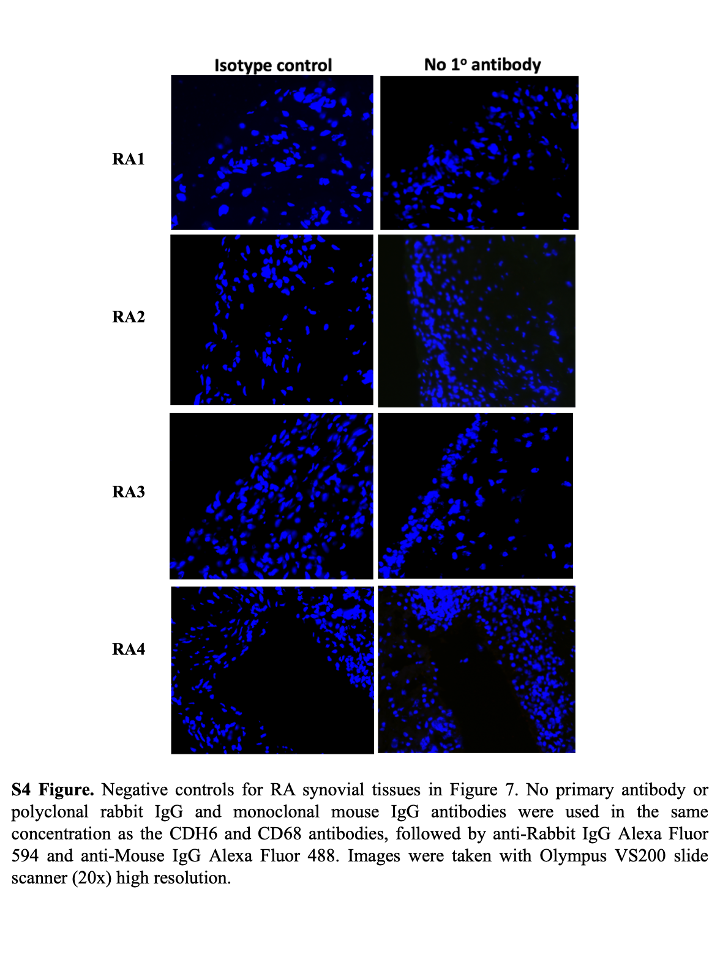

Supplement: Supplementary file 4 — Supplementary Material 4 [file 13075_2025_3637_MOESM4_ESM.tiff]

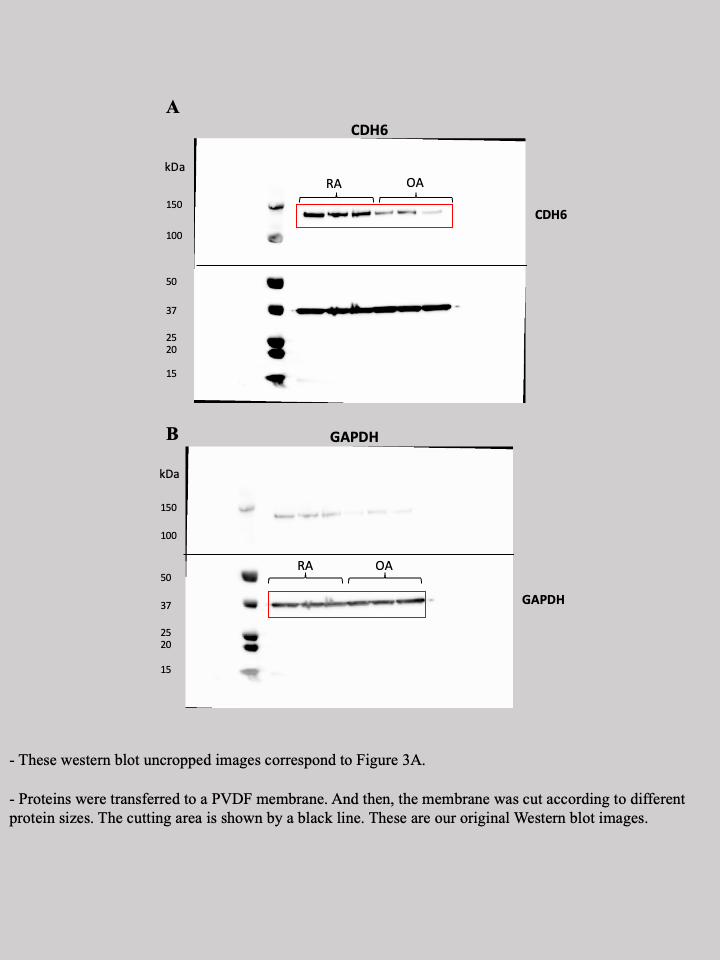

Supplement: Supplementary file 5 — Supplementary Material 5 [file 13075_2025_3637_MOESM5_ESM.tiff]

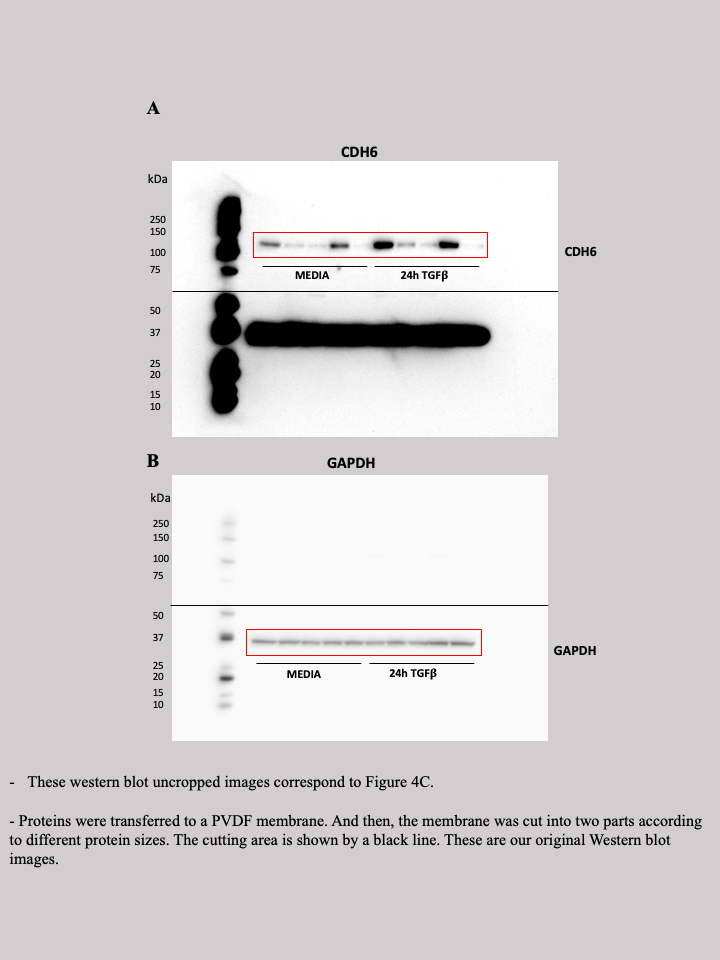

Supplement: Supplementary file 6 — Supplementary Material 6 [file 13075_2025_3637_MOESM6_ESM.tiff]

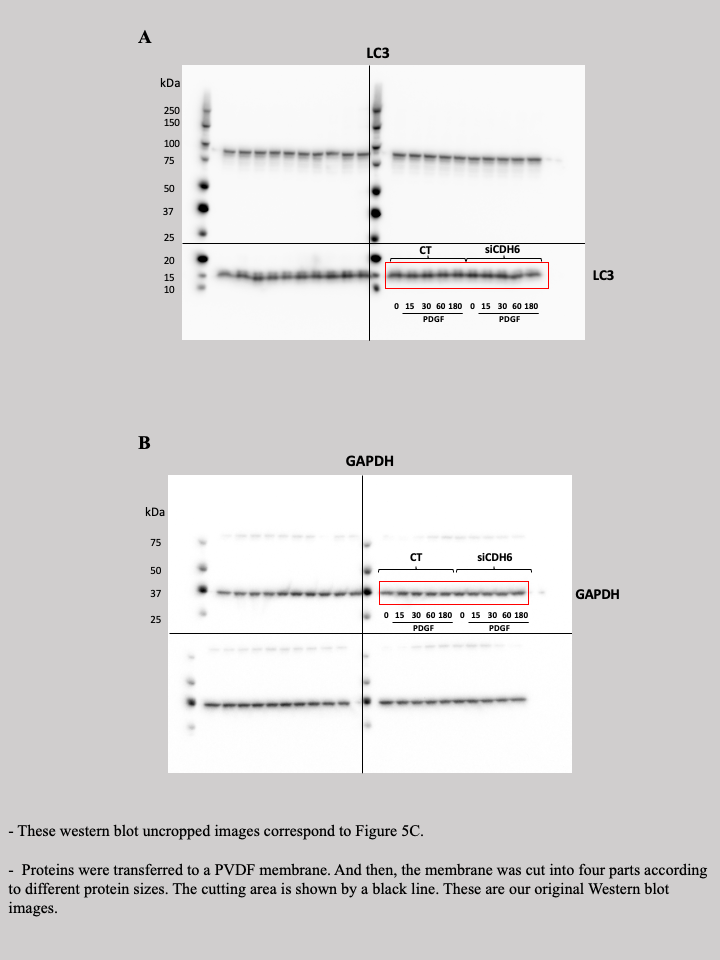

Supplement: Supplementary file 7 — Supplementary Material 7 [file 13075_2025_3637_MOESM7_ESM.tiff]

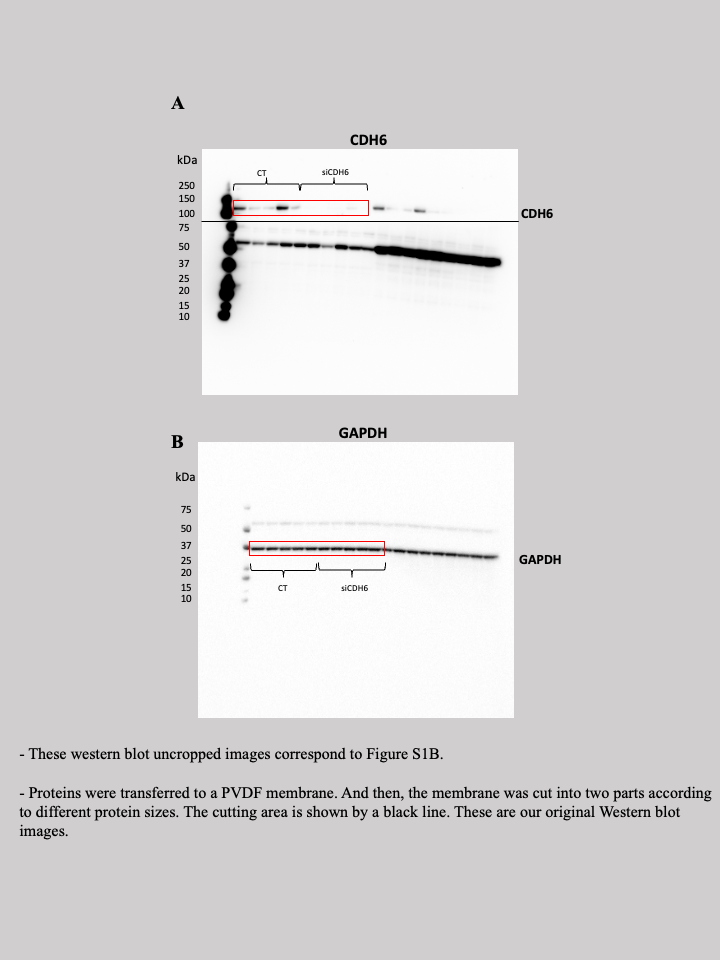

Supplement: Supplementary file 8 — Supplementary Material 8 [file 13075_2025_3637_MOESM8_ESM.tiff]

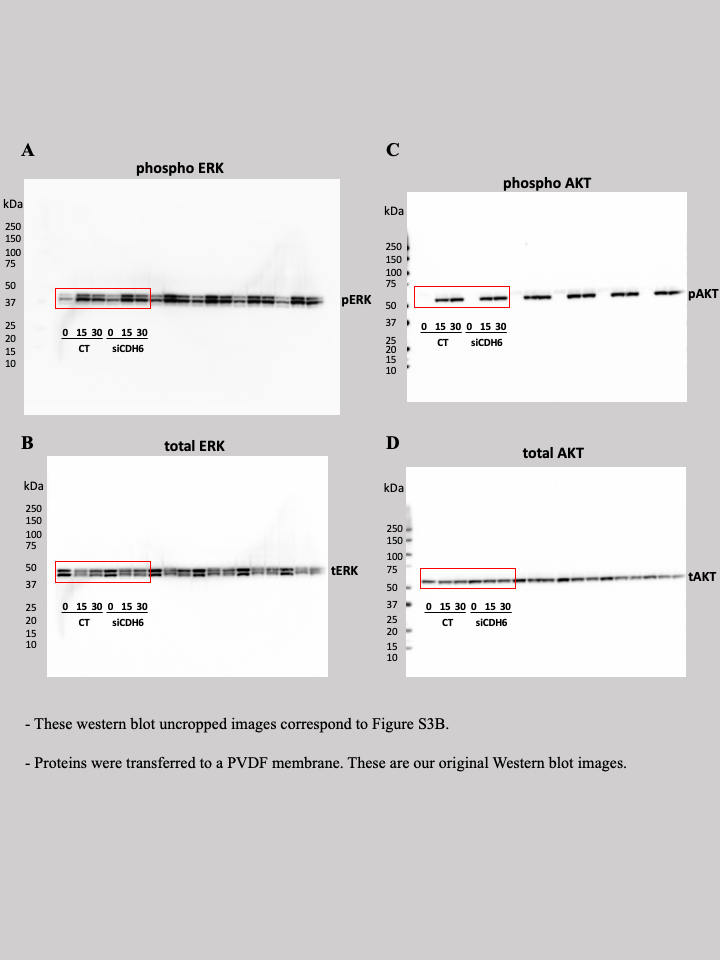

Supplement: Supplementary file 9 — Supplementary Material 9 [file 13075_2025_3637_MOESM9_ESM.tiff]

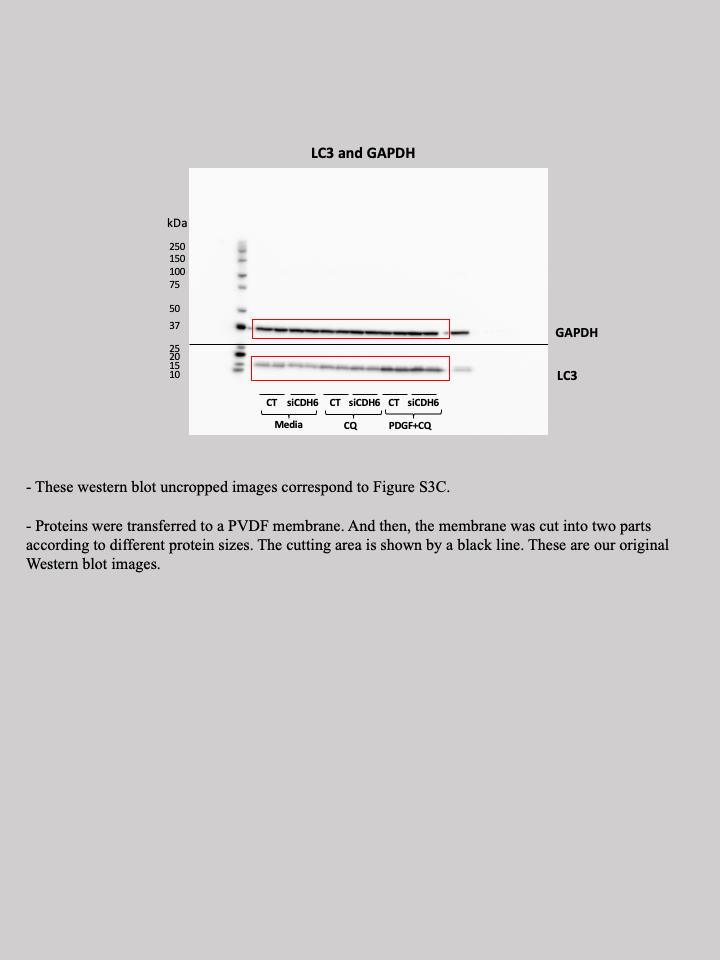

Supplement: Supplementary file 10 — Supplementary Material 10 [file 13075_2025_3637_MOESM10_ESM.tiff]
